# Effects of a One-Year Multicomponent Exercise Program on Community-Dwelling Older Adults at Risk of Sarcopenic Obesity

**DOI:** 10.3390/jcm14217839

**Published:** 2025-11-04

**Authors:** Alejandro Álvarez-Bustos, Samuel da Silva Aguiar, Ivan de Oliveira Gonçalves, Isabel Rodríguez-Sánchez, Emanuele Marzetti, Helio José Coelho-Junior

**Affiliations:** 1 Department of Health, Villanueva University, 28034 Madrid, Spain; a.alvarezbu@gmail.com; 2 Center for Proteomic and Biochemical Analysis, Post-Graduation in Genomic and Biotechnology Sciences, Catholic University of Brasilia, Brasilia 71966-700, Brazil; ssaguiar0@gmail.com; 3 Fit Senior–Center for Therapeutical Exercise, São Paulo 08557-400, Brazil; ivanogedfisica@gmail.com; 4 Instituto de Investigación Sanitaria Hospital Clínico San Carlos, 28040 Madrid, Spain; irsanchez@salud.madrid.org; 5 Fondazione Policlinico Universitario Agostino Gemelli IRCCS, 00168 Rome, Italy; 6 Department of Geriatrics, Orthopedics and Rheumatology, Università Cattolica del Sacro Cuore, 00168 Rome, Italy

**Keywords:** muscle strength, muscle power, mobility, exercise training

## Abstract

**Objectives:** The present study was conducted to examine the effects of a one-year multicomponent exercise training (MCET) program on the physical function and cardiovascular risk factors of community-dwelling older adults at risk of sarcopenic obesity. **Methods:** Data of 78 Brazilian community-dwelling older adults at risk of sarcopenic obesity, identified as the simultaneous presence of probable sarcopenia and overweight, were examined. The MCET program was performed twice a week over one year. Physical performance evaluations included (i) a timed “up-and-go” (TUG), (ii) one-leg stand, (iii) walking speed (WS) at normal pace and fast pace, (iv) a 5-time sit-to-stand (5STS) test, and (v) isometric handgrip strength (IHG). Cardiovascular risk factors involved blood pressure (BP) values and waist-to-hip ratio. **Results:** Significant improvements in balance and WS at a normal pace were observed following the MCET program, while no changes were noted in other physical performance markers. Additionally, a significant reduction in diastolic BP was recorded. **Conclusions:** Findings indicated significant improvements in mobility and balance, as well as a notable reduction in diastolic BP, among community-dwelling older adults at risk of sarcopenic obesity following a one-year MCET program. These improvements may play a critical role in reducing the risk of adverse outcomes such as falls, disability, cardiovascular events, hospitalization, and mortality. However, the quasi-experimental design of the present study, the absence of a control group, and other methodological limitations restrict the generalizability of the results. Future research using more rigorous study designs is necessary to confirm and expand upon these findings.

## 1. Introduction

Sarcopenic obesity (SO) refers to a condition characterized by the simultaneous occurrence of excessive adipose tissue and reduce muscle function and mass [1]. SO has emerged as a major public health problem because it exacerbates the risk of a wide range of adverse health outcomes. Indeed, individuals with SO are at heightened risk for both neuromuscular impairments, such as frailty and decreased mobility [2], and cardiometabolic diseases [3]. The combination of these factors not only compromises physical health and quality of life but also places a substantial burden on healthcare systems. As the global population ages [4], the prevalence of SO is expected to rise, making it critical to identify effective strategies for prevention and management [1].

Exercise training has been recognized as a promising strategy for managing SO [1,5]. Studies have found that resistance (RT) and aerobic training (AT) modalities [6,7,8,9] might improve neuromuscular and body composition markers in older adults with SO. However, these findings are not unanimous, as some studies have reported conflicting results [6,7,8,9]. Notably, exercise interventions targeting SO must stimulate adaptations across various physiological systems to be truly effective and potentially reverse this condition. In this regard, Multicomponent Exercise Training (MCET) stands out for its ability to simultaneously promote distinct modifications (e.g., increased muscle strength, reduced blood pressure [BP]), offering a comprehensive approach to improving both muscle and cardiometabolic health in individuals with SO [10,11].

However, although some investigations have combined RT and AT [6,7], to the best of our knowledge, no studies have examined the effects of MCET protocols on SO. Based on these premises, the present study was conducted to examine the effects of a one-year MCET intervention on the physical function and cardiovascular risk factors of community-dwelling at risk of sarcopenic obesity.

## 2. Materials and Methods

### 2.1. Study Design

This is a quasi-experimental study that examined the effects of a one-year MCET intervention on the physical function and cardiovascular markers of community-dwelling older adults at risk of SO. This study was approved by the Research Ethics Committee of the University of Mogi das Cruzes and was developed in accordance with the Declaration of Helsinki of the World Medical Association (1964, as revised in 1975, 1983, 1989, 1989, 1996 and 2000) and according to Resolution 196/96 of the National Health Council. Reporting of this study was guided by the TREND checklist [12], with selective application of relevant items (see Supplementary Materials S1).

### 2.2. Participants

Data examined in the present study were drawn from the Cantinho do Idoso da Cidade de Poá cohort [13,14,15]. This study, initiated in 2013 by Prof. Helio Jose Coelho Junior, Prof. Samuel da Silva Aguiar, and Prof. Ivan de Oliveira Gonçalves, aimed to assess, evaluate, compare, and describe the physical performance status of community-dwelling older adults attending a senior center in Poá, São Paulo, Brazil. Evaluations were conducted every six months until 2019.

Participants were recruited by convenience and asked verbally by the medical team and researchers about their participation in the study. Candidates were eligible to be part of the present study if they: (a) had at least 60 years of age, (b) were community-dwellers, (c) were independent to perform the activities of daily living, according to Katz Index (6 points) [16], (d) were able to ambulate independently without an assistive device, (e) had no dementia according to age- and schooling-adjusted Mini-Mental State Examination (MMSE) scores [17,18], and (f) signed the informed consent form. Candidate participants were excluded if they reported changes in pharmacological therapy during the investigation, experienced physical (e.g., angina) and/or psychological (e.g., fear) discomfort during exercise sessions, had a clinical diagnosis of pulmonary disease, neurological or psychiatric disease (e.g., Parkinson’s or Alzheimer’s disease), musculoskeletal disorders, experienced persistent dizziness, blurred vision or light-headedness when rising or standing for prolonged periods, and were absent from more than three physical exercise sessions. We also excluded participants prescribed hormone replacement therapy and/or psychotropic drugs.

For the current study, only data from participants at risk of SO in the 2014 evaluation with no missing values were analyzed (*n* = 78). These participants represent 13.9% of the total 2014 cohort (*n* = 562).

### 2.3. Risk of Sarcopenic Obesity

Risk of SO was defined by the presence of probable sarcopenia—operationalized as low isometric handgrip strength (IHG) and/or poor performance on the five-time sit-to-stand (5STS) test, as recommended by the European Working Group on Sarcopenia in Older People 2 (EWGSOP2) [19],—and a body mass index (BMI) ≥ 25 kg/m^2^, indicating overweight, as defined by the World Health Organization (WHO) [20].

### 2.4. Outcomes

This was an exploratory study aiming to assess the potential effects of exercise training across multiple health domains in older adults. Given its exploratory design, no single primary outcome was pre-specified. Study outcomes included physical performance and cardiovascular risk factors. Physical performance evaluations included (i) Timed “up-and-go” (TUG), (ii) one-leg stand, (iii) walking speed (WS) at normal pace and fast pace, (iv) 5STS test, and (v) IHG [21], while cardiovascular risk factors comprised BP and waist-to-hip ratio. Information regarding the psychometric properties of these instruments has been previously published [21].

#### 2.4.1. Timed “Up-and-Go”

The TUG test required participants to rise from a chair (overall height: 87 cm; seat height: 45 cm; width: 33 cm), walk 3 m around a floor marker, return to the starting point, and sit back down. Twenty-two participants completed the task wearing their usual shoes, with their backs resting against the backrest, arms placed on the armrests, and feet positioned on the ground. At the researcher’s signal (“go”), they stood up, walked 3 m at their fastest safe pace, turned around the cone, walked back, and sat down. The stopwatch started as soon as the participant rose from the chair and stopped once their back contacted the chair’s backrest again.

#### 2.4.2. One-Leg Stand

The one-leg stance test required participants to balance on their dominant leg while the opposite knee was bent at a 90° angle. During the test, their arms were crossed over the chest and the head remained upright. Timing started once the non-dominant foot was lifted from the ground and ended when it touched the floor again. A ceiling of 30 s was established as the maximum trial duration.

#### 2.4.3. Walking Speed at Normal Pace and Fast Pace

WS was assessed over a central 3-m distance. To complete the test, participants walked a total of 5 m, which included a 1-m zone for acceleration and another for deceleration. They were instructed to walk first at their normal pace and then as fast as possible without running. At the start, both feet had to be placed behind the initial line. Timing began when a foot crossed the 1-m mark and stopped once a foot reached the 4-m mark. The additional meter at each end of the walkway ensured that measurements were not influenced by starting acceleration or slowing down.

#### 2.4.4. Five-Time Sit-to-Stand

Participants rose from a chair 5 times as quickly as possible with their arms folded across their chest. Timing began when participants raised their buttocks off the chair and was stopped when they were seated at the end of the fifth stand.

#### 2.4.5. Isometric Handgrip Strength

IHG strength of the dominant hand was assessed with a Jamar hydraulic dynamometer (Sammons Preston, Bolingbrook, IL, USA). Dominance was determined by asking participants which hand they considered strongest. Measurements were taken with participants seated, shoulder slightly abducted, elbow flexed at 90° and held close to the torso, and wrist in a neutral “thumbs-up” position. The opposite arm was kept relaxed beneath the thigh. Each participant executed a maximal grip contraction lasting 4 s.

### 2.5. Cardiovascular Risk Factors

#### 2.5.1. Blood Pressure

BP measurements were conducted following guidelines adapted from the Seventh Report of the Joint National Committee on Prevention, Detection, Evaluation, and Treatment of High Blood Pressure (JNC 7) [22]. Prior to measurement, participants were asked to sit quietly in a comfortable chair within a calm environment for 15 min. A properly sized cuff—selected based on individual arm circumference (Sanny, São Paulo, Brazil)—was then placed at heart level on the upper left arm. An automated, noninvasive, and validated BP monitor (Microlife-BP 3BT0A, Microlife, Widnau, Switzerland) was used to assess both systolic (SBP) and diastolic BP (DBP) [23].

During the procedure, participants remained seated with feet flat and shoulder-width apart, backs supported, forearms resting on the table with palms facing upward, and were instructed to avoid movement or conversation. BP readings were taken three times, each separated by a one-minute rest interval, with each measurement lasting approximately 80 s. The average of the three readings was used for analysis. Participants were not informed of their BP values during the assessment.

#### 2.5.2. Waist-to-Hip Ratio

Waist circumference was measured at the midpoint between the lowest rib cage and the uppermost point of the iliac crest. Hip circumference was determined at the maximal protrusion of the buttocks.

### 2.6. Multicomponent Exercise Training

The MCET program was described according to the Template for Intervention, Description, and Replication (TIDieE) checklist (Supplementary Materials S2). And followed the framework proposed by Tarazona-Santabalbina et al. [24], which defines MCET as incorporating endurance, strength, coordination, balance, and flexibility exercises.

**Figure 1:**
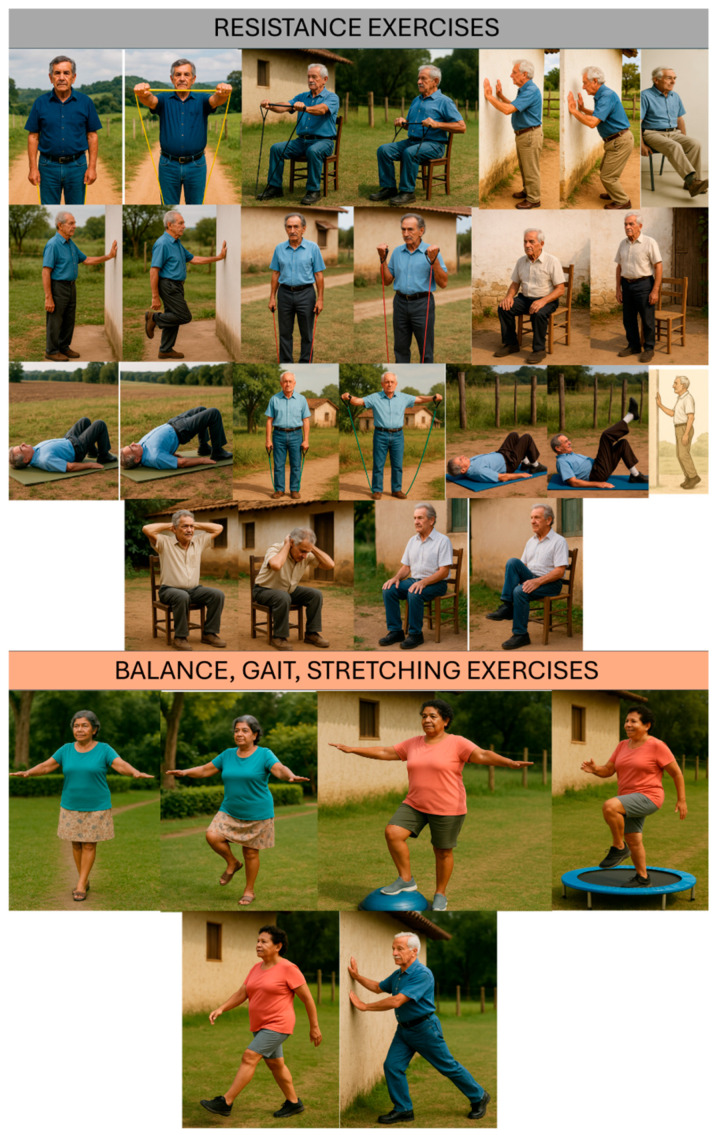
Examples of physical exercises used in the MCEP.

The training protocol consisted of 13 exercise stations, each including resistance, balance/proprioception, coordination, or flexibility work, along with gait tasks (endurance component). Examples of the physical exercises used in the present study are shown in Figure 1.

Specifically, six stations targeted resistance (five lower-limb and one upper-limb), four emphasized balance/proprioception, two focused on coordination, and one addressed flexibility. Each station was performed for one minute, while gait activities lasted two minutes.

Exercise intensity was monitored with the rating of perceived exertion (RPE) scale (adapted Borg CR-10) [25]. This scale ranges from 0 (rest) to 10 (maximal effort), with six verbal anchors (i.e., very, very easy; easy; moderate; somewhat hard; hard; very hard). Participants were instructed to maintain an intensity of 3–5, corresponding to moderate (3), somewhat hard (4), and hard (5), during functional (excluding balance) and resistance tasks (Table 1). To support self-monitoring, a large wall chart of the RPE scale (4 m high × 1.3 m wide) was displayed in the training area.

Progressive overload was introduced by adjusting exercise cadence and, for resistance training, by incorporating elastic bands (EXTEX Sports, São Paulo, Brazil) or dumbbells to achieve the prescribed exertion level.

Sessions lasted approximately 60 min, were held twice weekly on nonconsecutive days, and continued for one year. They were conducted face-to-face in groups under the supervision of exercise physiologists at a senior center in Poá, Brazil. Instructors ensured participants’ safety, proper execution of exercises, and also provided motivation throughout the sessions. As participants in the MCEP were expected to have preserved physical function, a standardized set of exercises was prescribed. However, slight modifications (e.g., reduced range of motion, slower walking pace, or omission of balance exercises) were made when participants reported acute conditions such as musculoskeletal pain.

### 2.7. Statistical Analysis

Continuous variables are presented as mean  ±  standard deviation (SD), whereas categorical variables are shown as absolute frequencies and percentages. Paired t-tests were conducted to assess differences in continuous measures. Bonferroni adjustment was applied to control for Type I error due to the presence of multiple comparisons. Cohen’s effect size *d* was calculated to assess the magnitude of the results. The effect size was classified as small (0.20–0.49), medium (0.50–0.79), large (≥0.80). Statistical significance was set at *p*  <  0.05 (two-tailed). All analyses were performed with SPSS software, version 23.0 (SPSS Inc., Chicago, IL, USA).

## 3. Results

No adverse events were reported during the exercise sessions or evaluations. All participants attended at least 80% of the scheduled sessions, with no participants dropping out. Adherence to the physical exercise program was 100%.

### 3.1. Characteristics of Study Participants

The main characteristics of the 78 study participants are presented in Table 2. The majority were men (73.1%), and the average age was 65.9 ± 5.4 years, indicating that the sample consisted of relatively young community-dwelling older adults.

### 3.2. Effects of MCET on Physical Performance and Cardiovascular Risk Markers

Table 3 presents the impact of the MCET on physical performance. Significant improvements in TUG (−7.0%), balance (+53.0%), and WS at normal pace (+12.6%) were observed following the MCET. After applying Bonferroni’s correction (see Supplementary Materials S3), only improvements in balance (*p* < 0.001), walking speed at normal pace (*p* < 0.001), and DBP (*p* < 0.001) remained significant. According to the effect size analysis, medium improvements were found in balance and WS, while TUG changes were small. DBP also showed a significant reduction (−3.1%) following MCET, with effect sizes indicating small changes. No statistically significant changes were seen for IHG (+2.1%), fast-pace WS (+20.7%), 5STS (+10%), SBP (−1.4%), or waist-to-hip ratio (−0.1%).

## 4. Discussion

Findings of the present study indicate significant improvements in mobility and balance, along with a significant reduction in DBP, among community-dwelling older adults at risk of sarcopenic obesity following a one-year MCET program.

An increasing number of studies have examined the effects of exercise training on SO, with findings that are often conflicting. These discrepancies seem to depend on specific characteristics of the interventions. Specifically, pooled analyses [6,7,8,9,26] suggest that muscle strength typically improves in response to RT, while no significant or small changes are seen with AT or combined RT and AT modalities [6,7,8,9]. On the other hand, mobility tends to improve with both RT and combined programs [6,7,9], although these results are not unanimous [8].

Findings of the present study are partially aligned with these investigations, given that significant improvements in balance (+53.0%) and WS performances (+12.6%) were observed following the MCET intervention. If confirmed in large randomized clinical trials, these observations would have important clinical implications, as the proposed low-cost exercise training program might be used as a strategy to reduce the risk of adverse events in community-dwelling older adults with SO. Indeed, improved performance in the physical function tests conducted in this study has been linked to a reduced risk of falls, disability, hospitalization, and death [27,28,29,30,31,32].

Furthermore, the average increase observed in WS (0.16 m/s) reached the minimal clinically important difference (MCID) [33], suggesting that the intervention may have contributed to meaningful improvements in mobility, with potential implications for daily functioning and reduced risk of adverse health outcomes. A subgroup analysis was conducted to examine how many participants experienced improvements equal or higher than the MCID (0.1 m/s) [33], with 32.1% meeting this threshold (Supplementary Materials S4).

However, the fact that other important physical performance markers associated with clinical outcomes were not improved after the MCET program indicates that some modifications might be necessary to enhance the intervention’s effectiveness. For example, gains in muscle strength are typically seen in response to exercise programs that involve frequent sessions each week, with a significant moderate-to-high intensity RT component [34,35]. Moreover, the lack of improvements in both WS at fast pace and 5STS likely indicates an insufficient power stimulus [36,37,38,39].

Muscle power refers to the capacity of generating strength quickly [40,41]. Improvements in muscle power are more likely to occur with exercise programs specifically designed to maximize the speed of concentric muscle contractions [42,43]. In light of this, expert consensus supports power training as an effective and valid approach to enhancing muscle power and physical performance in older adults [44,45]. Therefore, power training and high-intensity RT should be integrated into future studies examining the effects of MCET as an attempt to maximize improvements in physical performance.

A complementary observation is that participants of the present study had a relatively higher baseline average physical performance. Specifically, 5STS and WS at normal and fast pace were greater than age- and gender-specific normative values established for the Brazilian and Italian populations [21,46]. This scenario might indicate that very specific interventions, likely combining multiple tailored strategies (e.g., physical exercise, nutrition) would be necessary to promote further improvements in these measures. Furthermore, although only reported in distinct settings from that investigated in the present study [47], the possibility that improvements in 5STS in response to interventions are linked to a ceiling effect cannot be ruled out.

Regarding IHG, pooled analyses of the literature have raised the possibility that this performance measure has low responsiveness to interventions. The meta-analysis by Labott et al. [48] examined 24 trials involving over 3000 older adults and found that exercise training produced only modest improvements in IHG. Similarly, Grgic et al. [49] assessed the effects of RT on the IHG of very old adults (aged 75 years and above). Their findings indicated no statistically significant effect. In contrast, a meta-analysis by Chen et al. [50] reported a significant beneficial effect of RT on the IHG of older adults with sarcopenia. Nevertheless, due to substantial heterogeneity across studies (I^2^ = 81%), subgroup analyses were performed, revealing that significant improvements were limited to participants aged 70 years or younger, women, and those diagnosed according to the Asian Working Group for Sarcopenia (AWGS) consensus criteria. This aspect deserves to be better explored in future and specific investigations.

DBP was also significantly reduced following the MCET, suggesting that the intervention examined in this study may have substantially lowered the risk of major cardiovascular disease events, including coronary heart disease, stroke, and heart failure, as well as death [51]. This is particularly relevant given that individuals with SO face an elevated risk of developing cardiometabolic diseases [1,3].

These findings are consistent with a substantial body of literature highlighting the critical role of exercise training as a first-line strategy for managing BP in older adults. Cornelissen and Smart et al. [52] observed that exercise training interventions lasting at least 4 weeks significantly reduced BP in older adults, independent of the modality of exercise (e.g., AT, RT). Henkin et al. [53] expanded these results by providing a detailed examination of RT. Authors reported that interventions using exercise machines and dumbbells are more effective than those employing elastic bands, and that RT programs performed at low intensities are not significantly effective in lowering BP [53]. More recently, Coelho-Junior et al. [54] found that power training might significantly reduce resting BP levels in older adults.

Notably, the potential reductions promoted by MCET observed in the present study (~2.4 mmHg) are comparable to those found following well-established exercise strategies, including AT (2.5 mmHg), RT (−3.0 mmHg), combined AT and RT (2.5 mmHg), and high-intensity interval training (2.5 mmHg) [55]. These findings underscore the need for randomized controlled trials to further investigate the potential of MCET as a recommended therapy for managing BP. This is particularly important as it combines the reduction in the risk of both cardiovascular and neuromuscular issues, offering a holistic approach to improving health outcomes in older adults. Moreover, the dual benefits of MCET—enhancing both cardiovascular function and muscle strength—highlight its promise as an integrated intervention for managing age-related health challenges.

No differences were observed between in waist-to-hip circumference. Other studies have shown limited effects of exercise alone on body composition makers of older adults with SO [9,26] A possible explanation for this outcome is the absence of a combined nutritional intervention. In fact, incorporating dietary strategies alongside exercise has been shown to enhance the effectiveness of body composition improvements by supporting muscle growth, reducing fat mass, and optimizing metabolic health [9,26].

Proposing a nutritional strategy that contributes to reducing waist-to-hip circumference while preserving muscle mass and physical performance is complex and remains a major topic in health sciences [56]. Some authors have suggested that calorie-restricted diets, when combined with the intake of high-quality proteins—with or without supplementation of specific micronutrients (e.g., vitamin D)—may offer a promising approach [56,57]. However, further studies are needed to investigate this strategy, particularly in combination with MCET. Ideally, these investigations need to, along with an intervention based on MCET and diet, include a control group, and examine more clinically significant outcomes (e.g., falls, hospitalization, death).

The present study has several limitations that limit the generalizability of our results and should be considered to ensure a more accurate interpretation of the findings. First, as a quasi-experimental design, the study is limited by the absence of randomization, which can introduce selection biases and reduce the ability to establish causal relationships. Second, without a control group, it is difficult to definitively attribute any observed changes to the intervention itself, as external factors may have influenced the outcomes. Third, participants were recruited based on convenience, as it refers to an exploratory study. A primary outcome and an a priori calculation need to be performed in future investigations. Fourth, participants in the present study represent a specific population of independent community dwellers with high physical performance. The high attendance rate and lack of loss to follow-up reflects the fact that the exercise training protocol was provided by the municipal government, and participants were not allowed to be absent more than three times. This scenario suggests that extrapolations should be made with caution. Fifth, we excluded individuals who changed their pharmacological treatment during the study. This approach was taken to avoid potential confounding effects. Future studies should examine the impact of pharmacological therapy as a covariate. Sixth, important exercise variables, such as intensity and adherence, were not systematically controlled, which could have impacted the overall effectiveness of the intervention. Seventh, the lack of muscle mass evaluation hindered a more comprehensive assessment of sarcopenia status. Eighth, the study did not assess participants’ nutritional status or protein intake, both of which are key factors in muscle health and may have affected the results. Ninth, body composition measures were not included, limiting the ability to evaluate changes in fat and lean mass. Tenth, ambulatory BP values were not assessed, which could have provided more robust data on the impact of the intervention.

Finally, the potential biomarkers and mechanisms underlying the effectiveness of the MCEP were not examined. Specifically, oxidative stress, inflammatory markers, and adipokines are frequently associated with both sarcopenia and nutritional status [58,59,60], and could serve as a starting point for future studies.

## 5. Conclusions

The findings indicate significant improvements in mobility and balance, as well as a notable reduction in DBP, among community-dwelling older adults at risk of sarcopenic obesity following a one-year MCET program. These improvements may play a critical role in reducing the risk of adverse outcomes such as falls, disability, cardiovascular events, hospitalization, and mortality. However, the quasi-experimental design of the present study, the absence of a control group, and other methodological limitations restrict the generalizability of the results. Future research using more rigorous study designs is necessary to confirm and expand upon these findings. Ideally, these investigations need to, along with an intervention based on MCET and diet, include a control group, and examine more clinically significant outcomes (e.g., falls, hospitalization, death).

## Figures and Tables

**Table 1:** Rating Perceived Exertion scale.

RPE	Descriptor	Perceived Effort
0	Rest	No exertion at all
1	Very, very easy	Barely noticeable effort
2	Easy	Light effort
3	Moderate	Comfortable, steady effort
4	Somewhat hard	Challenging but sustainable
5	Hard	Strong effort, manageable
6–7	Very hard	Difficult to maintain
8–9	Extremely hard	Near-maximal effort
10	Maximal effort	Absolute exhaustion

**Table 2:** Main characteristics of study participants (*n* = 78).

Variable	Mean ± SD/%
Age (years)	65.9 ± 5.4
BMI (kg/m^2^)	29.35 ± 3.36
Sex (men)	57 (73.1)
Balance (s)	13.73 ± 11.98
TUG (s)	7.33 ± 1.45
WS at normal pace (m/s)	1.22 ± 0.25
WS at fast pace (m/s)	1.72 ± 0.61
IHG (kg)	19.65 ± 4.59
5STS (s)	11.06 ± 3.62
SBP (mmHg)	132.6 ± 18.5
DPB (mmHg)	76.6 ± 10.1
Waist-to-hip ratio	0.94 ± 0.41

5STS = 5-time sit-to-stand (5STS) test, BMI = Body mass index, IHG = Isometric handgrip; TUG = Timed “up-and-go”, WS = walking speed.

**Table 3:** Effects of MCET on physical function and cardiovascular markers.

Variable	Pre (Mean ± SD)	Post (Mean ± SD)	95% CIs	*Cohen’s d*
TUG (s)	7.33 ± 1.45	6.82 ± 1.62 *	0.15, 0.88	0.32 (small)
Balance (s)	13.73 ± 11.98	21.04 ± 10.03 *	−10.64, −4.66	0.66 (medium)
WS at normal pace (m/s)	1.22 ± 0.25	1.38 ± 0.21 *	0.23, 0.50	0.60 (medium)
WS at fast pace (m/s)	1.72 ± 0.61	1.79 ± 0.31	−0.01, 1.95	0.23 (small)
IHG (kg)	19.65 ± 4.59	20.06 ± 6.59	−2.40, 1.03	0.14 (negligible)
5STS (s)	11.06 ± 3.62	10.89 ± 5.09	−0.65, 2.13	0.12 (negligible)
SBP (mmHg)	132.6 ± 18.5	130.7 ± 17.6	−0.02, 3.76	0.09 (negligible)
DBP (mmHg)	76.6 ± 10.1	74.2 ± 9.6 *	1.38, 3.40	0.21 (small)
Waist-to-hip ratio	0.94 ± 0.41	0.94 ± 0.09	−0.04, 0.04	0.00 (negligible)

* *p* < 0.05 vs. Pre. **Abbreviation**: 5STS = 5-time sit-to-stand (5STS) test, DBP = Diastolic blood pressure; IHG = Isometric handgrip; SBP = Systolic blood pressure; TUG = Timed “up-and-go”; WS = walking speed.

## Data Availability

The datasets generated and analyzed during the current study are available from the corresponding senior authors upon reasonable request and subject to agreement.

## Supplementary Materials

The following supporting information can be downloaded at: https://www.mdpi.com/article/10.3390/jcm14217839/s1. Supplementary Materials S1. TREND Statement Checklist; Supplementary Materials S2. TIDieR Checklist; Supplementary Materials S3. Bonferroni’s adjustment; Supplementary Materials S4. Proportion of participants who reached the Minimal clinically important difference. Legend Supplementary Materials S4. MCID = Minimal clinically important difference.
